# Factors associated with intern noncompliance with the 2003 Accreditation Council for Graduate Medical Education’s 30-hour duty period requirement

**DOI:** 10.1186/1472-6920-12-33

**Published:** 2012-07-13

**Authors:** Christopher G Maloney, Armand H Matheny Antommaria, James F Bale, Jian Ying, Tom Greene, Rajendu Srivastava

**Affiliations:** 1 Department of Pediatrics, University of Utah School of Medicine, Salt Lake City, UT, USA; 2 Department of Biomedical Informatics, University of Utah School of Medicine, Salt Lake City, UT, USA; 3 Department of Internal Medicine, University of Utah School of Medicine, Salt Lake City, UT, USA; 4 Department of Neurology, University of Utah School of Medicine, Salt Lake City, UT, USA

## Abstract

**Background:**

In 2003 the Accreditation Council for Graduate Medical Education mandated work hour restrictions. Violations can results in a residency program being cited or placed on probation. Recurrent violations could results in loss of accreditation. We wanted to determine specific intern and workload factors associated with violation of a specific mandate, the 30-hour duty period requirement.

**Methods:**

Retrospective review of interns’ performance against the 30-hour duty period requirement during inpatient ward rotations at a pediatric residency program between June 24, 2008 and June 23, 2009. The analytical plan included both univariate and multivariable logistic regression analyses.

**Results:**

Twenty of the 26 (77%) interns had 80 self-reported episodes of continuous work hours greater than 30 hours. In multivariable analysis, noncompliance was inversely associated with the number of prior inpatient rotations (odds ratio: 0.49, 95% confidence interval (0.38, 0.64) per rotation) but directly associated with the total number of patients (odds ratio: 1.30 (1.10, 1.53) per additional patient). The number of admissions on-call, number of admissions after midnight and number of discharges post-call were not significantly associated with noncompliance. The level of noncompliance also varied significantly between interns after accounting for intern experience and workload factors. Subject to limitations in statistical power, we were unable to identify specific intern characteristics, such as demographic variables or examination scores, which account for the variation in noncompliance between interns.

**Conclusions:**

Both intern and workload factors were associated with pediatric intern noncompliance with the 30-hour duty period requirement during inpatient ward rotations. Residency programs must develop information systems to understand the individual and experience factors associated with noncompliance and implement appropriate interventions to ensure compliance with the duty hour regulations.

## Background

In 2003 the Accreditation Council for Graduate Medical Education (ACGME), in the U.S., implemented national resident duty hour requirements in response to concerns that excessive work hours were contributing to medical errors [1-3]. Major elements of the regulations included: a maximum work week of 80 hours averaged over four weeks, maximum in-house call frequency of every third night averaged over 4 weeks, and a maximum continuous duty period of 30 hours (24 hours with an additional 6 hours for transitional and educational activities) [1]. While acknowledging that these requirements can be difficult to monitor and enforce, the ACGME and residency review committees can impose citations, probation, or loss of accreditation on programs that fail to substantially comply with resident duty hour requirements.

There are some data in the literature regarding the frequency of residents violating the 2003 ACGME duty hour requirements [4-6]. For example, Landrigan and colleagues’ national prospective cohort study found that working shifts greater than 30 consecutive hours was the most common violation, reported by 67.4% of interns, and working more than 80 hours weekly averaged over 4 weeks was the second most common violation, reported by 43.0%. Little is known, however, about the specific factors that contribute to the violation of the extended duty periods.

At the University of Utah in Salt Lake City, UT, U.S., a review of the pediatric residency program revealed that the most frequent source, of duty-period violations, was interns (postgraduate year 1 (PGY1)) working more than 30 consecutive hours during their overnight in-house calls on the general inpatient ward rotation. The objective of this study was to determine specific intern and workload factors associated with violation of the 30-hour duty period requirement.

## Methods

### Setting

The study took place at Primary Children’s Medical Center in Salt Lake City, Utah, U.S. Primary Children’s is a 289 bed, freestanding children’s hospital owned and operated by Intermountain Healthcare. It is the primary teaching site of the University of Utah’s pediatric residency program. The program includes 59 categorical pediatric, 11 internal medicine-pediatric, and 10 pediatric-child psychiatry-adult psychiatry (triple board) residents.

### Study design

This study was a retrospective review from June 24, 2008 through June 23, 2009.

### Subjects

During the study period, interns performed two to five, four-week general inpatient rotations (four to five rotations for categorical pediatric interns, two for internal medicine-pediatric interns, and two for triple board interns). The inpatient teams were not differentiated in terms of ward location or specialty patients. Each team was comprised of a hospitalist teaching attending, a senior pediatric resident, two interns and intermittently, one third and one fourth year medical student. The attending of record for a given patient was categorized for the purposes of this study as community if the attending physician was also the primary care provider, hospitalist if the attending was the team hospitalist teaching attending or specialty if the attending was a sub-specialist. Each team generally capped at 16 patients. The team cap increased to 18 when a fourth year medical student (sub-intern) was assigned.

Interns took in-house, overnight call every fourth night in rotation with their teams. On call days, interns arrived at 0600, cared for existing patients during the day, and admitted new patients between 1600 and 0600. Admissions were capped at 10 per intern per night and admissions exceeding the team cap were transferred to other teams in the morning. The senior residents (PGY2 through PGY4) operated under a day-team/night-float system that utilized the team’s senior resident during the day (0700–1900) and a night-float team of two senior residents overnight (1900–0700).

### Study variables

We postulated that the following intern and workload factors might be related to noncompliance with the 30-hour duty period requirement:

Intern Factors: age (proxy for other work experience), marital status (proxy for support structure and/or commitments outside of training), pregnancy (self or spouse) or children (proxy for commitments outside of training), United States Medical Licensure Examination (USMLE) Step 1 and 2 scores (proxy for general knowledge), pediatric in-service training examination score (proxy for pediatric knowledge), and number of inpatient rotations (Rotations) (proxy for ward experience).

Workload Factors (per Intern): number of admissions (Total admissions, proxy for workload on call), number of admissions between midnight and 0600 (Post-midnight admissions, proxy for sleep on call), number of patients post-call (Total patients, proxy for workload post-call), number of hospitalist patients post-call (Hospitalist patients), number of non-hospitalist patients post-call (Non-hospitalist patients, combination of community and specialty categories, proxy for the number of different attendings the intern must contact post-call and workload post-call), and number of discharges on the post-call day (Discharges, proxy for workload post-call).

The primary outcome variable, noncompliance, was defined according to the ACGME requirements as reporting more than 30 continuous work hours.

### Data sources

Intern self-reported data on continuous work hours were obtained from the residency program. Interns reported duty-hours at the end of each rotation. Each rotation included 6 or 7 overnight, in-house calls. Intern factors were collected from the residency program database that routinely collects this information on all housestaff. Workload data was generated from an in-house clinical information system, Patient Tracker. Patient Tracker is used to manage patient flow and facilitate resident hand-off [7]. The interns and residents assign individual patients to their teams and specify the intern and attending categories using pull-down menus. Patient admission and discharge dates and times are automatically populated in Patient Tracker from the hospital admission-discharge-transfer (ADT) database table [8].

### Analytical plan

#### Univariate analyses relating reported noncompliance to predictor variables

Separate generalized linear mixed effects logistic regression models [9,10] were used to relate reported noncompliance to the predictor variables. For this objective each potential predictor was analyzed individually, without adjustment for the other predictor variables. In each analysis, team was controlled as a potential confounder and intern was treated as a random effect. Odds ratios (adjusted for team) and their associated 95% confidence intervals and p-values are reported for each predictor variable.

#### Multivariable analyses relating reported noncompliance to predictor variables

Generalized multiple linear logistic mixed effects regression models were then used to jointly relate reported noncompliance to the following five predictors: Rotations, Post-midnight admissions, Hospitalist patients, Non-hospitalist patients, and Discharges. Team was again controlled as a potential confounder and intern was treated as a random effect. The variable, Total admissions, was not included in the multivariable models due to moderate collinearity with Post-midnight admissions (Pearson R = 0.55), with an associated variance inflation factor of 1.43. The highest Pearson R among the factors included in the multivariable model was 0.45 (variance inflation factor = 1.25), between Post-midnight admissions and Total patients. Total patients, Hospitalist patients, and Non-hospitalist patients were also mathematically related to each other. Therefore, linear contrasts were constructed to estimate adjusted odds ratios for reported noncompliance associated with changes in the numbers of Hospitalist and Non-hospitalist patients. The first three contrasts examined the addition of one patient to the team: adding one Hospitalist patient with no change in the number of Non-hospitalist patients; adding one Non-hospitalist patient with no change in the number of Hospitalist patients, and adding one ‘average’ patient with equal weight given to Hospitalist and Non-hospitalist patients. A fourth contrast evaluated keeping the total team size fixed but replacing one Non-hospitalist patient with one Hospitalist patient.

In exploratory analyses, the multivariable models were expanded to include individual intern characteristics in addition to the specified workload predictors to investigate if these factors might explain intern-variation in reported noncompliance rates. Hypothesis tests were regarded as statistically significant if p < 0.05 without adjustment for multiple comparisons.

The University of Utah Institutional Review Board and the Intermountain Healthcare Privacy Board both approved this study.

## Results

There were 26 total interns enrolled in the residency program during the study period. Their mean (standard deviation, SD) age was 28.3 (3.3) years, 20 (77%) were female, 18 (69%) were single, and 5 (19%) became pregnant during their intern year or already had children. Their mean USMLE-1 score was 224 (21), mean USMLE-2 score was 238 (22) and their mean in-service training exam score was 176 (120). The National five-year moving average of in-service training exam score was 148.

Table 1 summarizes the characteristics, by intern, of the 728 post-call days during the study period. The number of rotations ranged from 2 to 5, with a mean of 2.7 (1.4). The mean number of patients on the post-call day was 6.8 (1.9), which included 3.8 (1.7) hospitalist and 3.0 (1.6) non-hospitalists patients. Twenty interns (77%) reported a total of 80 episodes of continuous work hours that were greater than 30 hours.

**Table 1:** Summary of Workload Factors (per intern)

Variable (N = 728)	Mean (SD)	10% Percentile	50% percentile	90% percentile
Rotations	2.7 (1.4)	1	3	5
Post-Midnight Admissions	1.2 (1.0)	0	1	2
Total Admissions	3.4 (1.5)	2	3	5
Hospitalist Patients	3.8 (1.7)	2	4	6
Non-Hospitalist Patients	3.0 (1.6)	1	3	5
Total Patients	6.8 (1.9)	4	7	9
Discharges	1.4 (1.2)	0	1	3

Table 2 provides the results of univariate analyses relating reported noncompliance to seven designated predictor variables while controlling only for the team factor. The number of prior rotations was associated with decreased noncompliance, while greater numbers of Total patients and of Non-hospitalist patients were each associated with increased noncompliance.

**Table 2:** Univariate Analyses Relating Reported Noncompliance to Predictor Variables^*^

Predictor Variable (N = 728)	Odds Ratio
Rotations	0.51 (0.40, 0.66) (p < 0.001)
Admissions	1.25 (0.98, 1.58) (p = 0.07)
Total Admissions	1.10 (0.92, 1.31) (p = 0.31)
Hospitalist Patients	1.03 (0.88, 1.20) (p = 0.73)
Non-Hospitalist Patients	1.18 (1.01, 1.37) (p = 0.04)
Total Patients	1.18 (1.02, 1.36) (p = 0.03)
Discharges	0.89 (0.71, 1.11) (p = 0.30)

^*^Shown are odds ratios for noncompliance, 95% confidence intervals, and p-values. All odds ratios are adjusted for team.

Table 3 summarizes the results of the multivariable analyses relating reported noncompliance to the following workload factors: the numbers of Rotations, Post-midnight admissions, Discharges, and Total patients. After controlling for the other factors in the model, each additional rotation was associated with a 51% (36%, 62%) reduction in the odds of noncompliance. If equal weights are given to Hospitalist and Non-hospitalist patients, each increase in team size by 1 patient was associated with a 30% (10%, 53%) increase in the odds of noncompliance. No significant effect on reported noncompliance was observed if “Total patients” was fixed and one Non-hospitalist patient was exchanged for one Hospitalist patient. Numbers of Post-midnight admissions and Discharges were not significantly associated with reported noncompliance.

**Table 3:** Multivariable Analyses Relating Reported Noncompliance to Predictor Variables^*^

Predictor Variable (N = 728)	Adjusted Odds Ratio
Rotations	0.49 (0.38,0.64) (p < 0.001)
Admissions after Midnight	1.15 (0.89, 1.47) (p = 0.29)
Increase Team Size by 1 Hospitalist Patient	1.25 (1.03,1.52) (p = 0.03)
Increase Team Size by 1 Non-Hospitalist Patient	1.34 (1.11,1.62) (p = 0.003)
Increase Team Size by 1 Patient	1.30 (1.10,1.53) (p = 0.003)
Fix Team Size and Exchange 1 Non-Hospitalist for Hospitalist Patient	0.94 (0.78,1.13) (p = 0.49)
Discharges	0.86 (0.67,1.10) (p = 0.24)

^*^Shown are adjusted odds ratios, 95% confidence intervals and p value for the fixed effects. All odds ratios are adjusted for team in addition to the factors listed in the table. The odds of compliance varied significantly among interns after adjustment for the factors in the table (p = 0.03).

After accounting for the workload factors listed in Table 3, noncompliance probabilities also differed significantly among the 26 interns (p = 0.03).

Exploratory analyses relating noncompliance rates to either intern age, presence of pregnancy or children, or to the three examination scores found that none of these factors was significantly related to noncompliance after controlling for the other systems factors in the model.

A sensitivity analysis that added duty hour reporting of exactly 30 hours (n = 198) to the noncompliant category demonstrated similar results (Data not shown).

## Discussion

We found that 20 (77%) interns had 80 episodes of continuous duty hour reporting greater than 30 hours. The intern factor associated with reported noncompliance was the number of inpatient rotations (a strong inverse association). Noncompliance varied significantly among interns after accounting for the workload factors. The workload factors associated with reported noncompliance were the number of Non-hospitalist patients and increasing total patients (modest positive associations). The number of admissions on-call, number of admissions after midnight, total patients post-call and number of discharges post-call were not found to be significant risk factors for noncompliance.

We designed this study to inform the modification of intern workflow to ensure compliance with the 2003 ACGME duty hour requirements in the U.S. Based on the preliminary analysis of this data, the pediatric residency program decreased the cap on overnight admission in July 2010 from 8 to 6 patients per intern for the first 2 call nights of the intern’s first ward rotation. The ACGME, however, has eliminated extended duty periods for interns, effective July 1, 2011[11]. The residency program then reduced the maximum number of patients each intern could round on each day during the first week of his/her first ward rotation. The housestaff has not, however, reported this change to be helpful. While residents beyond their intern year are permitted to take overnight call, their workflow can differ from that of the interns. The method and results of this study, nonetheless, have important implications for this revised system and perhaps similar systems in other countries.

Residents will still need to comply with duty hour restrictions and residency programs will need to determine what factors contribute to noncompliance. There is limited literature on this topic. This may be due, in part, to the fact that collecting data on resident workload is difficult, especially if it is dependent on residents maintaining patient logs. Data was available for this study from software designed, in part, to facilitate resident hand-offs, which is integrated into their workflow [7]. Many residency programs, including a recent large-scale collaborative research study [12,13], are focusing on hand-off communication as a way to decrease medical errors that may result from the increased number of hand-offs. Data systems to facilitate hand-off communication could be developed to capture granular data, which could be used for other purposes such as studying factors associated with noncompliance.

The identification of specific intern skills and attributes associated with compliance is a very important area of future study. Our results demonstrate that experience was associated with a larger reduction in the risk of noncompliance than other system factors. Noncompliance also varied significantly among the individual interns after accounting for the workload factors. These skills and attributes may not, however, be related to cognitive knowledge but skills such as organization and efficiency. There is, unfortunately, limited literature on resident productivity and the available literature is mainly focused on the emergency department [14]. If specific skills and attributes can be identified, the next question is whether they can be effectively taught thereby reducing noncompliance or permitting residents to carry larger patient loads.Hospitalists may be well suited to teach “efficiency” to housestaff. Hospitalists, both adult and pediatric, have reduced length of stay/costs between 10 – 15%. Hospitalist-educators are a growing group in academic centers who could focus on how best to teach efficiency to housestaff [15].

Our study has certain limitations. The current study relied on duty hour self-reporting, in a single-center and a single intern class. Duty hour reporting for the interns occurred at the end of each rotation creating the possibility of recall bias. Residents may underreport noncompliance [16]. However, our sensitivity analysis, which attempted to mitigate the potential for this bias, demonstrated similar results. The sample size limited the number of potential contributing factors that could be evaluated. For example, we did not include attending characteristics in our model. The sample size also limited the power to detect individual intern factors associated with noncompliance.

## Conclusions

Our study identified intern and workload factors associated with pediatric intern noncompliance with the 30-hour duty period requirement during general inpatient ward rotations. The number of general inpatient rotations demonstrated the largest effect and characteristics of the intern him/herself were also associated with noncompliance. Contrary to expectations, other variables such as number of admissions after midnight and number of discharges on the post-call day were not associated with the risk of noncompliance. The results of this study suggest that programs must identify the intern and system characteristics associated with noncompliance and identify the optimum educational strategies necessary to enhance intern and resident compliance with duty hour regulations.

## Competing interests

The authors of this manuscript have no competing interests or potential conflicts of interest, financial or other, to disclose. The authors previously presented this material as a poster at the Pediatric Academic Societies Annual Meeting, May 2010, in Vancouver, British Columbia, Canada.

## Authors’ contributions

CGM contributed in conception, design, data acquisition, analysis and interpretation of data, drafted and revised the manuscript and approved the final version. AHMA contributed in conception, design, interpretation of data, revised the manuscript and approved the final version. JFB contributed in design, data acquisition, interpretation of data, revised the manuscript and approved the final version. JY contributed in analysis, revised the manuscript and approved the final version. TG contributed in design, analysis and interpretation of data, revised the manuscript and approved the final version. RS contributed in conception, design, interpretation of data, revised the manuscript and approved the final version. All authors read and approved the final manuscript.

## Authors’ information

CGM MD PhD is Division Chief for Pediatric Inpatient Medicine and professor, Departments of Pediatrics and Biomedical Informatics, University of Utah School of Medicine.

AHMA MD PhD is Chair of the Biomedical Ethics Program at Primary Children’s Medical Center in Salt Lake City and associate professor, Departments of Pediatrics and Internal Medicine, University of Utah School of Medicine.

JFB MD is the pediatric residency program director and professor, Departments of Pediatrics and Neurology, University of Utah School of Medicine.

JY PhD is a research associate in the Study Design and Biostatistics Center, Department of Internal Medicine, University of Utah School of Medicine

TG PhD is the Director of the Study Design and Biostatics Center in the Division of Epidemiology and professor, Department of Internal Medicine, University of Utah School of Medicine.

RS MD FRCP(C) MPH is the current Chairperson of the International, Pediatric Research in Inpatient Settings (PRIS) Network and associate professor, Department of Pediatrics, University of Utah School of Medicine

## Pre-publication history

The pre-publication history for this paper can be accessed here:

http://www.biomedcentral.com/1472-6920/12/33/prepub
